# PDA: Pooled DNA analyzer

**DOI:** 10.1186/1471-2105-7-233

**Published:** 2006-04-28

**Authors:** Hsin-Chou Yang, Chia-Ching Pan, Chin-Yu Lin, Cathy SJ Fann

**Affiliations:** 1 Institute of Biomedical Sciences, Academia Sinica, Nankang, Taipei, 115, Taiwan

## Abstract

**Background:**

Association mapping using abundant single nucleotide polymorphisms is a powerful tool for identifying disease susceptibility genes for complex traits and exploring possible genetic diversity. Genotyping large numbers of SNPs individually is performed routinely but is cost prohibitive for large-scale genetic studies. DNA pooling is a reliable and cost-saving alternative genotyping method. However, no software has been developed for complete pooled-DNA analyses, including data standardization, allele frequency estimation, and single/multipoint DNA pooling association tests. This motivated the development of the software, 'PDA' (Pooled DNA Analyzer), to analyze pooled DNA data.

**Results:**

We develop the software, PDA, for the analysis of pooled-DNA data. PDA is originally implemented with the MATLAB^® ^language, but it can also be executed on a Windows system without installing the MATLAB^®^. PDA provides estimates of the coefficient of preferential amplification and allele frequency. PDA considers an extended single-point association test, which can compare allele frequencies between two DNA pools constructed under different experimental conditions. Moreover, PDA also provides novel chromosome-wide multipoint association tests based on p-value combinations and a sliding-window concept. This new multipoint testing procedure overcomes a computational bottleneck of conventional haplotype-oriented multipoint methods in DNA pooling analyses and can handle data sets having a large pool size and/or large numbers of polymorphic markers. All of the PDA functions are illustrated in the four bona fide examples.

**Conclusion:**

PDA is simple to operate and does not require that users have a strong statistical background. The software is available at .

## Background

The millions of single nucleotide polymorphisms (SNPs) now available are ideal for association analyses that identify important genetic variants in populations as well as genes predisposed to diseases involving complex traits [1,2]. Although the cost of individual genotyping has been reduced drastically over the years, the use of DNA pooling has reduced the cost even further, especially for large-scale studies. The first DNA pooling study was performed to identify the association between HLA class II loci and disease genes predisposing type 1 diabetes [3]. DNA pooling was later used to estimate the allele frequency of short tandem repeats and SNPs, map disease susceptibility genes [4,5], and identify polymorphisms [6-8]. A comprehensive review of the history of DNA pooling, the methods and algorithms involved, and the application thereof can refer to [9] and [10].

DNA pooling is highly efficient. Many researchers have investigated the performance of DNA pools while estimating allele frequency and have measured the impact of pooling on association test results. The results show that allele frequencies can be estimated accurately and precisely using DNA pools after considering coefficient of preferential amplification (CPA) [11,12]; moreover, the test power is high and the false-positive rate is well controlled [11,13]. These promising results suggest that DNA pooling studies is reliable and cost-saving relative to individual genotyping studies. This motivated the development of the software, Pooled DNA Analyzer (PDA), to analyze pooled DNA data.

Although many single-point pooled DNA association tests have been developed, multipoint analysis still presents a challenge due to the large numbers of genotypic combinations in DNA pools. The difficulty increases substantially with the pool size and/or the number of SNPs involved. Several of the recently proposed advanced multipoint estimations and tests have been haplotype oriented [14-17]; nevertheless, all such methods require a small pool size and a small number of SNPs to reduce both the computational complexity and running time. To address the current computational challenges of analyzing DNA pools, PDA provides the sliding-window empirical p-value test (SWEPT), which has advantages with respect to statistical computation, data implementation and practical application. The SWEPT method is particularly applicable when the analysis involves a large amount of data, which overcomes the computational bottleneck of conventional haplotype-oriented multipoint methods in DNA pooling analyses.

## Implementation

**Figure 1:**
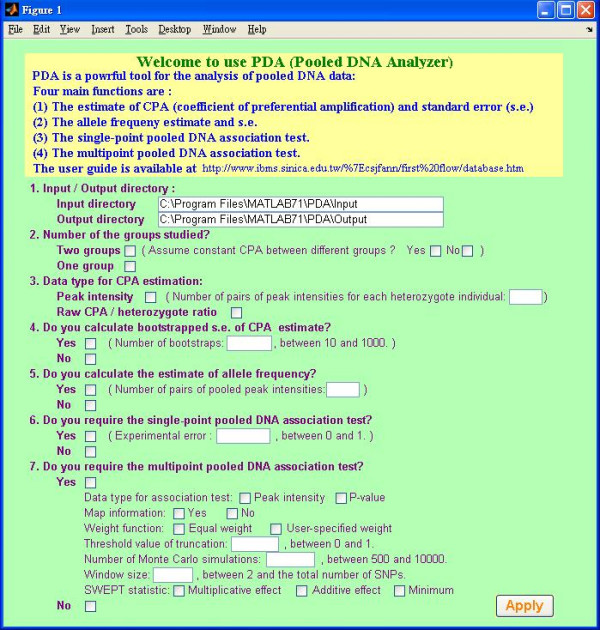
Interface of PDA.

PDA was developed on the MATLAB^® ^software platform that is adapted to the Windows systems (MS Windows^® ^98/ME and MS Windows^® ^NT/2000/XP/2003). For MATLAB^® ^users, PDA can be run with a graphical user-friendly interface where users merely click the checkboxes to carry out data analysis. The PDA user interface is shown in Figure 1. For those who have no access to or little knowledge of the MATLAB^® ^system, we used the MATLAB^® ^compiler to generate standalone executables of PDA, which can be deployed on machines without installing the MATLAB^®^. The guide to the installation and initialization of PDA on Windows is illustrated in Appendix A (See Additional File 1). Description of working directories for PDA is shown in Appendix B (See Additional File 2). The PDA's input and output data formats are explained in Appendices C and D (See Additional files 3 and 4), respectively. Finally, the compiled version of PDA is demonstrated in Appendix E (See Additional File 5).

### Interface of PDA, item functions and operation procedures

There are seven main items in the PDA menu, i.e., input/output directory, number of groups studied, data type for CPA estimation, bootstrapped standard error (s.e.) of CPA estimates, allele frequency estimates, single-point pooled DNA association test and multipoint pooled DNA association test.

Item 1. Input/Output directory: The directories of input and output files must be specified. PDA will read data from the assigned input directory and automatically save outputs in the output directory. The format of input and output is illustrated in Appendices C and D (See Additional files 3 and 4).

Item 2. Number of groups studied: PDA can analyze one-group or two-group DNA pooling data. For one-group studies, users can estimate CPA and calculate adjusted allele frequency by checking the box 'One group'. For two-group studies (e.g., case control studies), users check the box 'Two groups' and determine whether to carry out association tests after calculating estimates for CPA and allele frequency. PDA provides the flexibility of equal or unequal CPA statistical inference that the user may choose as needed. Check 'Yes' for equal CPA inference or 'No' for unequal CPA inference.

Item 3. Data type for CPA estimation: Two types of data are acceptable. The first type is peak intensity data from genotyping experiments. The second type is raw CPA/heterozygote ratio from empirical studies or databases. If peak intensity data are inputted, then users should provide the number of pairs of peak intensities for each locus.

Item 4. Calculation of the bootstrapped s.e. of the CPA estimate: Bootstrapping is a resampling technique used to estimate the s.e. of CPA. Users can determine whether s.e. is to be calculated. If users want to calculate the bootstrapped s.e. then they should check 'Yes' and assign a number of bootstrap replications between 10 and 1000. A larger number of bootstrap replications will take longer to calculate but yields a more reliable estimate.

Item 5. Estimation of adjusted allele frequency: Users can check 'Yes' to calculate the adjusted allele frequencies or 'No' to omit the calculation.

Item 6. Single-point pooled DNA association test: Users can carry out association tests only for the analysis of a two-group study. Because the test statistic of association tests depends on experimental error, users must assign a proper value for the experimental standard error, *σ*_E_, if an association test is conducted.

Item 7. Multipoint pooled DNA association test: Users can carry out association tests only for the analysis of a two-group study. If they check 'Yes', they must answer seven options to conduct this test. The seven options are as follows. (1) Data type for the association test. Two types of data are acceptable: peak intensity data or raw p-values from previous single-point association tests. (2) Map information. Users can check 'Yes' to provide information on marker positions for the latter graph demonstration of multipoint p-values or check 'No' to ignore the inter-marker distances. (3) Weight function. Users can choose to assign equal weights to all marker loci by checking 'Equal weight' or provide a set of weights by checking 'User-specified weight'. (4) Threshold value of truncation. PDA provides a function to truncate insignificant p-values in the analysis. The value is between 0 and 1, and p-values greater than the threshold will be excluded from the analysis. (5) Number of Monte Carlo simulations. Users must provide a suitable number of simulations between 500 and 10000. A large number of simulations increase the accuracy of the empirical p-value estimation, but a longer computational time may be required. (6) Window size, defined as the number of markers in a window prior to p-value truncation. Users should specify a suitable number of markers in a window according to the attributes of their data. Window size must be = 2, with the upper limit being the total number of SNPs in the study. (7) SWEPT statistics. PDA provides three statistics for multipoint association tests; i.e., multiplicative, additive and minimum p-value statistics.

The statistical theory is introduced in the next section.

## Results

### Methodology

We developed PDA based on a four-stage procedure, which combines the concept of a three-stage DNA pooling experiment [11] with the procedure of a novel multipoint association test, SWEPT. The functions make PDA useful for a complete analysis of pooled DNA data.

Firstly, PDA provides estimates for the CPA, which affects allele frequency estimation and association testing in a pooled DNA study. For a diallelic SNP with alleles *A *and *a*, CPA represents the relative magnitude of the averaged amplified intensities of the different alleles and is defined mathematically as *κ *= *μ*_*A*_/*μ*_*a*_, where *μ*_*A *_and *μ*_*a *_are the average peak intensities of alleles *A *and *a*. The parameters can be estimated from heterozygous individuals who provide a standard for a 50:50 ratio for a pair of peak intensities of two heterozygous alleles. When *κ *= 1, there is no preferential amplification; when *κ *> 1, the first allele is more likely to be amplified than the second; when *κ *< 1, the second allele is more likely to be amplified than the first. PDA provides three discrete estimates for the CPA: arithmetic mean adjustment κ^H
 MathType@MTEF@5@5@+=feaafiart1ev1aaatCvAUfKttLearuWrP9MDH5MBPbIqV92AaeXatLxBI9gBaebbnrfifHhDYfgasaacH8akY=wiFfYdH8Gipec8Eeeu0xXdbba9frFj0=OqFfea0dXdd9vqai=hGuQ8kuc9pgc9s8qqaq=dirpe0xb9q8qiLsFr0=vr0=vr0dc8meaabaqaciaacaGaaeqabaqabeGadaaakeaaiiGacuWF6oWAgaqcamaaBaaaleaacqqGibasaeqaaaaa@2FB8@, unbiased adjustment κ^U
 MathType@MTEF@5@5@+=feaafiart1ev1aaatCvAUfKttLearuWrP9MDH5MBPbIqV92AaeXatLxBI9gBaebbnrfifHhDYfgasaacH8akY=wiFfYdH8Gipec8Eeeu0xXdbba9frFj0=OqFfea0dXdd9vqai=hGuQ8kuc9pgc9s8qqaq=dirpe0xb9q8qiLsFr0=vr0=vr0dc8meaabaqaciaacaGaaeqabaqabeGadaaakeaaiiGacuWF6oWAgaqcamaaBaaaleaacqqGvbqvaeqaaaaa@2FD2@ and geometric mean adjustment κ^G
 MathType@MTEF@5@5@+=feaafiart1ev1aaatCvAUfKttLearuWrP9MDH5MBPbIqV92AaeXatLxBI9gBaebbnrfifHhDYfgasaacH8akY=wiFfYdH8Gipec8Eeeu0xXdbba9frFj0=OqFfea0dXdd9vqai=hGuQ8kuc9pgc9s8qqaq=dirpe0xb9q8qiLsFr0=vr0=vr0dc8meaabaqaciaacaGaaeqabaqabeGadaaakeaaiiGacuWF6oWAgaqcamaaBaaaleaacqqGhbWraeqaaaaa@2FB6@ along with the corresponding bootstrap standard errors [11]. Let *n*_heter _denote the number of heterozygous individuals and {hAI
 MathType@MTEF@5@5@+=feaafiart1ev1aaatCvAUfKttLearuWrP9MDH5MBPbIqV92AaeXatLxBI9gBaebbnrfifHhDYfgasaacH8akY=wiFfYdH8Gipec8Eeeu0xXdbba9frFj0=OqFfea0dXdd9vqai=hGuQ8kuc9pgc9s8qqaq=dirpe0xb9q8qiLsFr0=vr0=vr0dc8meaabaqaciaacaGaaeqabaqabeGadaaakeaacqWGObaAdaqhaaWcbaGaemyqaeeabaacbaGae8xsaKeaaaaa@305D@(*j*), haI
 MathType@MTEF@5@5@+=feaafiart1ev1aaatCvAUfKttLearuWrP9MDH5MBPbIqV92AaeXatLxBI9gBaebbnrfifHhDYfgasaacH8akY=wiFfYdH8Gipec8Eeeu0xXdbba9frFj0=OqFfea0dXdd9vqai=hGuQ8kuc9pgc9s8qqaq=dirpe0xb9q8qiLsFr0=vr0=vr0dc8meaabaqaciaacaGaaeqabaqabeGadaaakeaacqWGObaAdaqhaaWcbaGaemyyaegabaacbaGae8xsaKeaaaaa@309D@(*j*), *j *= 1,...,*n*_heter_} is the pair of peak intensities of heterozygous individuals derived from individual genotypings. The mathematical formulas of the three CPA estimators are presented as follows:

κ^H=nheter-1×∑j=1nheter[hAI(j)/haI(j)],κ^U=κ^H+nheternheter−1(h¯AIh¯aI−κ^H),κ^G=(∏j=1nheterhAI(j)haI(j))nheter,
 MathType@MTEF@5@5@+=feaafiart1ev1aaatCvAUfKttLearuWrP9MDH5MBPbIqV92AaeXatLxBI9gBaebbnrfifHhDYfgasaacH8akY=wiFfYdH8Gipec8Eeeu0xXdbba9frFj0=OqFfea0dXdd9vqai=hGuQ8kuc9pgc9s8qqaq=dirpe0xb9q8qiLsFr0=vr0=vr0dc8meaabaqaciaacaGaaeqabaqabeGadaaakeaafaqabeWabaaabaacciGaf8NUdSMbaKaadaWgaaWcbaGaeeisaGeabeaakiabg2da9iabd6gaUnaaDaaaleaacqqGObaAcqqGLbqzcqqG0baDcqqGLbqzcqqGYbGCaeaacqqGTaqlcqqGXaqmaaGccqGHxdaTdaaeWbqaaiabcUfaBjabdIgaOnaaDaaaleaacqWGbbqqaeaacqqGjbqsaaGcdaqadaqaaiabdQgaQbGaayjkaiaawMcaaiabc+caViabdIgaOnaaDaaaleaacqWGHbqyaeaacqqGjbqsaaGcdaqadaqaaiabdQgaQbGaayjkaiaawMcaaiabc2faDjabcYcaSaWcbaGaemOAaOMaeyypa0JaeGymaedabaGaemOBa42aaSbaaWqaaiabbIgaOjabbwgaLjabbsha0jabbwgaLjabbkhaYbqabaaaniabggHiLdaakeaacuWF6oWAgaqcamaaBaaaleaacqqGvbqvaeqaaOGaeyypa0Jaf8NUdSMbaKaadaWgaaWcbaGaeeisaGeabeaakiabgUcaRmaalaaabaGaemOBa42aaSbaaSqaaiabbIgaOjabbwgaLjabbsha0jabbwgaLjabbkhaYbqabaaakeaacqWGUbGBdaWgaaWcbaGaeeiAaGMaeeyzauMaeeiDaqNaeeyzauMaeeOCaihabeaakiabgkHiTiabigdaXaaadaqadaqaamaalaaabaGafmiAaGMbaebadaqhaaWcbaGaemyqaeeabaGaeeysaKeaaaGcbaGafmiAaGMbaebadaqhaaWcbaGaemyyaegabaGaeeysaKeaaaaakiabgkHiTiqb=P7aRzaajaWaaSbaaSqaaiabbIeaibqabaaakiaawIcacaGLPaaacqGGSaalaeaacuWF6oWAgaqcamaaBaaaleaacqqGhbWraeqaaOGaeyypa0ZaaOqaaeaadaqadaqaamaarahabaWaaSaaaeaacqWGObaAdaqhaaWcbaGaemyqaeeabaGaeeysaKeaaOWaaeWaaeaacqWGQbGAaiaawIcacaGLPaaaaeaacqWGObaAdaqhaaWcbaGaemyyaegabaGaeeysaKeaaOWaaeWaaeaacqWGQbGAaiaawIcacaGLPaaaaaaaleaacqWGQbGAcqGH9aqpcqaIXaqmaeaacqWGUbGBdaWgaaadbaGaeeiAaGMaeeyzauMaeeiDaqNaeeyzauMaeeOCaihabeaaa0Gaey4dIunaaOGaayjkaiaawMcaaaWcbaGaemOBa42aaSbaaWqaaiabbIgaOjabbwgaLjabbsha0jabbwgaLjabbkhaYbqabaaaaOGaeiilaWcaaaaa@B084@

where h¯AI=nheter−1⋅∑j=1nheterhAI(j)
 MathType@MTEF@5@5@+=feaafiart1ev1aaatCvAUfKttLearuWrP9MDH5MBPbIqV92AaeXatLxBI9gBaebbnrfifHhDYfgasaacH8akY=wiFfYdH8Gipec8Eeeu0xXdbba9frFj0=OqFfea0dXdd9vqai=hGuQ8kuc9pgc9s8qqaq=dirpe0xb9q8qiLsFr0=vr0=vr0dc8meaabaqaciaacaGaaeqabaqabeGadaaakeaacuWGObaAgaqeamaaDaaaleaacqWGbbqqaeaacqqGjbqsaaGccqGH9aqpcqWGUbGBdaqhaaWcbaGaeeiAaGMaeeyzauMaeeiDaqNaeeyzauMaeeOCaihabaGaeyOeI0IaeGymaedaaOGaeyyXIC9aaabmaeaacqWGObaAdaqhaaWcbaGaemyqaeeabaGaeeysaKeaaOWaaeWaaeaacqWGQbGAaiaawIcacaGLPaaaaSqaaiabdQgaQjabg2da9iabigdaXaqaaiabd6gaUnaaBaaameaacqqGObaAcqqGLbqzcqqG0baDcqqGLbqzcqqGYbGCaeqaaaqdcqGHris5aaaa@5268@ and h¯aI=nheter−1⋅∑j=1nheterhaI(j)
 MathType@MTEF@5@5@+=feaafiart1ev1aaatCvAUfKttLearuWrP9MDH5MBPbIqV92AaeXatLxBI9gBaebbnrfifHhDYfgasaacH8akY=wiFfYdH8Gipec8Eeeu0xXdbba9frFj0=OqFfea0dXdd9vqai=hGuQ8kuc9pgc9s8qqaq=dirpe0xb9q8qiLsFr0=vr0=vr0dc8meaabaqaciaacaGaaeqabaqabeGadaaakeaacuWGObaAgaqeamaaDaaaleaacqWGHbqyaeaacqqGjbqsaaGccqGH9aqpcqWGUbGBdaqhaaWcbaGaeeiAaGMaeeyzauMaeeiDaqNaeeyzauMaeeOCaihabaGaeyOeI0IaeGymaedaaOGaeyyXIC9aaabmaeaacqWGObaAdaqhaaWcbaGaemyyaegabaGaeeysaKeaaOWaaeWaaeaacqWGQbGAaiaawIcacaGLPaaaaSqaaiabdQgaQjabg2da9iabigdaXaqaaiabd6gaUnaaBaaameaacqqGObaAcqqGLbqzcqqG0baDcqqGLbqzcqqGYbGCaeqaaaqdcqGHris5aaaa@52E8@. For each SNP, the estimated CPA will inform users of the magnitude of the difference in amplification between two alleles.

Secondly, PDA provides adjusted estimates for allele frequencies and the standard errors corresponding to the three different CPAs. Let κ^
 MathType@MTEF@5@5@+=feaafiart1ev1aaatCvAUfKttLearuWrP9MDH5MBPbIqV92AaeXatLxBI9gBaebbnrfifHhDYfgasaacH8akY=wiFfYdH8Gipec8Eeeu0xXdbba9frFj0=OqFfea0dXdd9vqai=hGuQ8kuc9pgc9s8qqaq=dirpe0xb9q8qiLsFr0=vr0=vr0dc8meaabaqaciaacaGaaeqabaqabeGadaaakeaaiiGacuWF6oWAgaqcaaaa@2E75@ be the estimated CPA. The adjusted allele frequency of allele *A *is estimated by p^A=hA/(hA+κ^×ha)
 MathType@MTEF@5@5@+=feaafiart1ev1aaatCvAUfKttLearuWrP9MDH5MBPbIqV92AaeXatLxBI9gBaebbnrfifHhDYfgasaacH8akY=wiFfYdH8Gipec8Eeeu0xXdbba9frFj0=OqFfea0dXdd9vqai=hGuQ8kuc9pgc9s8qqaq=dirpe0xb9q8qiLsFr0=vr0=vr0dc8meaabaqaciaacaGaaeqabaqabeGadaaakeaacuWGWbaCgaqcamaaBaaaleaacqWGbbqqaeqaaOGaeyypa0JaemiAaG2aaSbaaSqaaiabdgeabbqabaGccqGGVaWldaqadaqaaiabdIgaOnaaBaaaleaacqWGbbqqaeqaaOGaey4kaSccciGaf8NUdSMbaKaacqGHxdaTcqWGObaAdaWgaaWcbaGaemyyaegabeaaaOGaayjkaiaawMcaaaaa@3FAB@, where *h*_*A *_and *h*_*a *_denote the peak intensity of alleles *A *and *a *in a DNA pool [12]. These analyses can be applied to studies of a single group or two groups, and the information will help users understand the genetic distribution of their groups.

Thirdly, PDA provides a single-point association mapping of two groups (e.g., case control studies or comparative studies of two groups). Let *n*_G1 _and *n*_G2 _be the numbers of individuals in groups G1 and G2; κ^G1
 MathType@MTEF@5@5@+=feaafiart1ev1aaatCvAUfKttLearuWrP9MDH5MBPbIqV92AaeXatLxBI9gBaebbnrfifHhDYfgasaacH8akY=wiFfYdH8Gipec8Eeeu0xXdbba9frFj0=OqFfea0dXdd9vqai=hGuQ8kuc9pgc9s8qqaq=dirpe0xb9q8qiLsFr0=vr0=vr0dc8meaabaqaciaacaGaaeqabaqabeGadaaakeaaiiGacuWF6oWAgaqcamaaBaaaleaacqqGhbWrcqaIXaqmaeqaaaaa@30A6@ and κ^G2
 MathType@MTEF@5@5@+=feaafiart1ev1aaatCvAUfKttLearuWrP9MDH5MBPbIqV92AaeXatLxBI9gBaebbnrfifHhDYfgasaacH8akY=wiFfYdH8Gipec8Eeeu0xXdbba9frFj0=OqFfea0dXdd9vqai=hGuQ8kuc9pgc9s8qqaq=dirpe0xb9q8qiLsFr0=vr0=vr0dc8meaabaqaciaacaGaaeqabaqabeGadaaakeaaiiGacuWF6oWAgaqcamaaBaaaleaacqqGhbWrcqaIYaGmaeqaaaaa@30A8@ are the estimated CPAs in groups G1 and G2; D=p^AG1−p^AG2
 MathType@MTEF@5@5@+=feaafiart1ev1aaatCvAUfKttLearuWrP9MDH5MBPbIqV92AaeXatLxBI9gBaebbnrfifHhDYfgasaacH8akY=wiFfYdH8Gipec8Eeeu0xXdbba9frFj0=OqFfea0dXdd9vqai=hGuQ8kuc9pgc9s8qqaq=dirpe0xb9q8qiLsFr0=vr0=vr0dc8meaabaqaciaacaGaaeqabaqabeGadaaakeaacqWGebarcqGH9aqpcuWGWbaCgaqcamaaDaaaleaacqWGbbqqaeaacqqGhbWrcqaIXaqmaaGccqGHsislcuWGWbaCgaqcamaaDaaaleaacqWGbbqqaeaacqqGhbWrcqaIYaGmaaaaaa@3928@ denotes the difference of the estimated allele frequencies of allele *A *between two groups. The test statistic of single-point association mapping with adjustment for preferential amplification is X=D2/V^(D)
 MathType@MTEF@5@5@+=feaafiart1ev1aaatCvAUfKttLearuWrP9MDH5MBPbIqV92AaeXatLxBI9gBaebbnrfifHhDYfgasaacH8akY=wiFfYdH8Gipec8Eeeu0xXdbba9frFj0=OqFfea0dXdd9vqai=hGuQ8kuc9pgc9s8qqaq=dirpe0xb9q8qiLsFr0=vr0=vr0dc8meaabaqaciaacaGaaeqabaqabeGadaaakeaacqWGybawcqGH9aqpcqWGebardaahaaWcbeqaaiabikdaYaaakiabc+caViqbdAfawzaajaWaaeWaaeaacqWGebaraiaawIcacaGLPaaaaaa@35EA@, where the estimated variance is

V^(D)=p^AG1p^aG12nG1+p^AG2p^aG22nG2+[V^(κ^G1)1/2⋅p^AG1p^aG1κ^G1−V^(κ^G2)1/2⋅p^AG2p^aG2κ^G2]2+2σ^E2,
 MathType@MTEF@5@5@+=feaafiart1ev1aaatCvAUfKttLearuWrP9MDH5MBPbIqV92AaeXatLxBI9gBaebbnrfifHhDYfgasaacH8akY=wiFfYdH8Gipec8Eeeu0xXdbba9frFj0=OqFfea0dXdd9vqai=hGuQ8kuc9pgc9s8qqaq=dirpe0xb9q8qiLsFr0=vr0=vr0dc8meaabaqaciaacaGaaeqabaqabeGadaaakeaacuWGwbGvgaqcamaabmaabaGaemiraqeacaGLOaGaayzkaaGaeyypa0ZaaSaaaeaacuWGWbaCgaqcamaaDaaaleaacqWGbbqqaeaacqqGhbWrcqaIXaqmaaGccuWGWbaCgaqcamaaDaaaleaacqWGHbqyaeaacqqGhbWrcqaIXaqmaaaakeaacqaIYaGmcqWGUbGBdaWgaaWcbaGaee4raCKaeGymaedabeaaaaGccqGHRaWkdaWcaaqaaiqbdchaWzaajaWaa0baaSqaaiabdgeabbqaaiabbEeahjabikdaYaaakiqbdchaWzaajaWaa0baaSqaaiabdggaHbqaaiabbEeahjabikdaYaaaaOqaaiabikdaYiabd6gaUnaaBaaaleaacqqGhbWrcqaIYaGmaeqaaaaakiabgUcaRmaadmaabaGafmOvayLbaKaadaqadaqaaGGaciqb=P7aRzaajaWaaSbaaSqaaiabbEeahjabigdaXaqabaaakiaawIcacaGLPaaadaahaaWcbeqaaiabigdaXiabc+caViabikdaYaaakiabgwSixpaalaaabaGafmiCaaNbaKaadaqhaaWcbaGaemyqaeeabaGaee4raCKaeGymaedaaOGafmiCaaNbaKaadaqhaaWcbaGaemyyaegabaGaee4raCKaeGymaedaaaGcbaGaf8NUdSMbaKaadaWgaaWcbaGaee4raCKaeGymaedabeaaaaGccqGHsislcuWGwbGvgaqcamaabmaabaGaf8NUdSMbaKaadaWgaaWcbaGaee4raCKaeGOmaidabeaaaOGaayjkaiaawMcaamaaCaaaleqabaGaeGymaeJaei4la8IaeGOmaidaaOGaeyyXIC9aaSaaaeaacuWGWbaCgaqcamaaDaaaleaacqWGbbqqaeaacqqGhbWrcqaIYaGmaaGccuWGWbaCgaqcamaaDaaaleaacqWGHbqyaeaacqqGhbWrcqaIYaGmaaaakeaacuWF6oWAgaqcamaaBaaaleaacqqGhbWrcqaIYaGmaeqaaaaaaOGaay5waiaaw2faamaaCaaaleqabaGaeGOmaidaaOGaey4kaSIaeGOmaiJaf83WdmNbaKaadaqhaaWcbaGaeeyraueabaGaeeOmaidaaOGaeiilaWcaaa@8E9E@

where V^(κ^G1)
 MathType@MTEF@5@5@+=feaafiart1ev1aaatCvAUfKttLearuWrP9MDH5MBPbIqV92AaeXatLxBI9gBaebbnrfifHhDYfgasaacH8akY=wiFfYdH8Gipec8Eeeu0xXdbba9frFj0=OqFfea0dXdd9vqai=hGuQ8kuc9pgc9s8qqaq=dirpe0xb9q8qiLsFr0=vr0=vr0dc8meaabaqaciaacaGaaeqabaqabeGadaaakeaacuWGwbGvgaqcamaabmaabaacciGaf8NUdSMbaKaadaWgaaWcbaGaee4raCKaeeymaedabeaaaOGaayjkaiaawMcaaaaa@3377@ and V^(κ^G2)
 MathType@MTEF@5@5@+=feaafiart1ev1aaatCvAUfKttLearuWrP9MDH5MBPbIqV92AaeXatLxBI9gBaebbnrfifHhDYfgasaacH8akY=wiFfYdH8Gipec8Eeeu0xXdbba9frFj0=OqFfea0dXdd9vqai=hGuQ8kuc9pgc9s8qqaq=dirpe0xb9q8qiLsFr0=vr0=vr0dc8meaabaqaciaacaGaaeqabaqabeGadaaakeaacuWGwbGvgaqcamaabmaabaacciGaf8NUdSMbaKaadaWgaaWcbaGaee4raCKaeeOmaidabeaaaOGaayjkaiaawMcaaaaa@3379@ are the bootstrapped variances of the estimated CPAs in groups G1 and G2, and σ^E
 MathType@MTEF@5@5@+=feaafiart1ev1aaatCvAUfKttLearuWrP9MDH5MBPbIqV92AaeXatLxBI9gBaebbnrfifHhDYfgasaacH8akY=wiFfYdH8Gipec8Eeeu0xXdbba9frFj0=OqFfea0dXdd9vqai=hGuQ8kuc9pgc9s8qqaq=dirpe0xb9q8qiLsFr0=vr0=vr0dc8meaabaqaciaacaGaaeqabaqabeGadaaakeaaiiGacuWFdpWCgaqcamaaBaaaleaacqqGfbqraeqaaaaa@2FC3@ is the experimental standard error which can be estimated by calculating the root mean square error based on a hierarchical experimental design [18] or calculating the square root of variance components relied on the restricted maximum likelihood method [19]. The asymptotic distribution of test statistic *X *is a chi square distribution with one degree of freedom. This test reduces to the single-point association test proposed in [11] if the equality of CPAs in two groups is held. The test statistic and p-value are calculated and used to identify important SNPs. Association studies that compare more than two groups can be further analyzed by combining pair-wise analyses with multiple testing correction.

Fourthly, PDA provides a multipoint association test. A sliding-window empirical p-value method is introduced into pooled DNA analysis. Define {*v*_1_,...,*v*_*N*_} to be a p-value vector of *N *SNPs from single-point association tests, and the locations of SNPs follow the order of genetic or physical mappings. Let *k *denote the size of a sliding window. The SWEPT statistics, based on multiplicative and additive models in the *i*th window with window size *k*, are represented as follows: for *i *= 1,...,*N *+ 1 - *k*,

ZM(i,k)=∏j=ii+k−1vjwij×I[vj<μ],
MathType@MTEF@5@5@+=feaafiart1ev1aaatCvAUfKttLearuWrP9MDH5MBPbIqV92AaeXatLxBI9gBaebbnrfifHhDYfgasaacH8akY=wiFfYdH8Gipec8Eeeu0xXdbba9frFj0=OqFfea0dXdd9vqai=hGuQ8kuc9pgc9s8qqaq=dirpe0xb9q8qiLsFr0=vr0=vr0dc8meaabaqaciaacaGaaeqabaqabeGadaaakeaacqWGAbGwdaWgaaWcbaGaemyta0eabeaakmaabmaabaGaemyAaKMaeiilaWIaem4AaSgacaGLOaGaayzkaaGaeyypa0ZaaebmaeaacqWG2bGDdaqhaaWcbaGaemOAaOgabaGaem4DaC3aaSbaaWqaaiabdMgaPjabdQgaQbqabaWccqGHxdaTcqWGjbqscqGGBbWwcqWG2bGDdaWgaaadbaGaemOAaOgabeaaliabgYda8GGaciab=X7aTjabc2faDbaaaeaacqWGQbGAcqGH9aqpcqWGPbqAaeaacqWGPbqAcqGHRaWkcqWGRbWAcqGHsislcqaIXaqma0Gaey4dIunakiabcYcaSaaa@5455@

and ZA(i,k)=∑j=ii+k−1wij×vj×I[vj<μ],
 MathType@MTEF@5@5@+=feaafiart1ev1aaatCvAUfKttLearuWrP9MDH5MBPbIqV92AaeXatLxBI9gBaebbnrfifHhDYfgasaacH8akY=wiFfYdH8Gipec8Eeeu0xXdbba9frFj0=OqFfea0dXdd9vqai=hGuQ8kuc9pgc9s8qqaq=dirpe0xb9q8qiLsFr0=vr0=vr0dc8meaabaqaciaacaGaaeqabaqabeGadaaakeaacqWGAbGwdaWgaaWcbaGaemyqaeeabeaakmaabmaabaGaemyAaKMaeiilaWIaem4AaSgacaGLOaGaayzkaaGaeyypa0ZaaabmaeaacqWG3bWDdaWgaaWcbaGaemyAaKMaemOAaOgabeaakiabgEna0kabdAha2naaBaaaleaacqWGQbGAaeqaaOGaey41aqRaemysaKKaei4waSLaemODay3aaSbaaSqaaiabdQgaQbqabaGccqGH8aapiiGacqWF8oqBcqGGDbqxaSqaaiabdQgaQjabg2da9iabdMgaPbqaaiabdMgaPjabgUcaRiabdUgaRjabgkHiTiabigdaXaqdcqGHris5aOGaeiilaWcaaa@5675@

where *μ *is the threshold of the p-value truncation and *I*[*A*] is the usual indicator that takes the value of 1 if event *A *is true; otherwise, it takes the value of 0. The non-negative *w*_*ij *_is a standardized weight of the p-value, *v*_*j*_, in the *i*th window (i.e. the weight satisfies the requirement that the weights in the window sum to one). The standardized weight is calculated by dividing the original weight by the sum of all original weights in the window under the given original weights. The multiplicative SWEPT statistic is a sliding-window extension of the truncated product method [20], and the additive SWEPT statistic is an extension of the test statistic [21]. The third statistic is the minimum p-value in the window as follows:

*Z*_*Min*_(*i*,*k*) = min_*j *= *i*,...,*i*+*k*-1_{*v*_*j*_}, *i *= 1,...,*N *+ 1 - *k*.

The minimum SWEPT statistic extended the technique of taking the minimum score, which has good performances in test power and type 1 error and has been used broadly in genetic studies [22,23].

There are other efficient p-value combinations, such as the rank truncated product method [24], which may be considered in PDA in the future. Extension of these methods using sliding windows will help screen important genetic markers in large-scale chromosome-wide pooled DNA association studies. By default, PDA performs multipoint analysis by using p-value data obtained from the proposed single-point association; however, PDA also provides options for the use of p-value data yielded from other single-point methods.

To assess the statistical significance of the SWEPT in each window, PDA applied a Monte-Carlo procedure recommended in [20] to calculate an empirical p-value. The procedure generates the correlated p-value vector *V *with a correlation matrix ∑ from an independent p-value vector *V*_0_, based on the following correlation-invariant transformation

*V *= 1 - Φ(*C*^-1^Φ^-1^(1 - *V*_0_)),

where Φ(.) is the cumulative distribution of a standard normal random variable and *C *is a lower triangular matrix satisfying the Cholesky decomposition, ∑ = *CC*^*T*^. We estimated the correlation matrix ∑ using an autocorrelation function of p-values. We recalculated the SWEPT statistics based on the generated p-value vector, *V*. The previous procedure was repeated *B *times to yield {*Z*^(*b*)^(*i*, *k*), *b *= 1,...,*B*}. Hence, the empirical p-value of the *i*th window with window size *k *can be calculated as the following:

EP=∑b=1BI[Z(b)(i,k)<Z*(i,k)]/B,
 MathType@MTEF@5@5@+=feaafiart1ev1aaatCvAUfKttLearuWrP9MDH5MBPbIqV92AaeXatLxBI9gBaebbnrfifHhDYfgasaacH8akY=wiFfYdH8Gipec8Eeeu0xXdbba9frFj0=OqFfea0dXdd9vqai=hGuQ8kuc9pgc9s8qqaq=dirpe0xb9q8qiLsFr0=vr0=vr0dc8meaabaqaciaacaGaaeqabaqabeGadaaakeaacqWGfbqrcqWGqbaucqGH9aqpdaaeWaqaaiabdMeajjabcUfaBjabdQfaAnaaCaaaleqabaWaaeWaaeaacqWGIbGyaiaawIcacaGLPaaaaaGcdaqadaqaaiabdMgaPjabcYcaSiabdUgaRbGaayjkaiaawMcaaiabgYda8iabdQfaAjabcQcaQmaabmaabaGaemyAaKMaeiilaWIaem4AaSgacaGLOaGaayzkaaGaeiyxa0Laei4la8IaemOqaiealeaacqWGIbGycqGH9aqpcqaIXaqmaeaacqWGcbGqa0GaeyyeIuoakiabcYcaSaaa@4E65@

where Z*(*i,k*) is the corresponding SWEPT value based on real data. The SWEPT offers several advantages over conventional DNA pooling analyses. (1) SWEPT can work well even in cases where only p-value data are available; hence, it can analyze data from different study designs and is applicable to meta-analysis. Because SWEPT allows a p-value truncation, it also handles data containing unpublished insignificant p-values. (2) The SWEPT statistics make adjustments for preferential amplification, a critical aspect that has never been considered before in pooled DNA multipoint analyses. (3) The simplicity of the SWEPT statistics lowers processing time and significantly reduces the computational complexity. (4) The SNPs involved in multipoint analyses can be determined conveniently once the window size has been determined, thereby avoiding the common perplexity of selecting SNPs in haplotype-oriented or other multipoint analyses. (5) SWEPT is comprehensive in that it covers conventional single-point test statistics and can be applied to the analysis of individual genotyping data, although this aspect is not the primary concern of PDA.

### Real data analysis

We give four examples to illustrate functions of PDA: (1) One-group allele frequency estimation. (2) Two-group single-point DNA pooling studies. (3) Two-group multipoint association test based on peak intensity data. (4) Two-group multipoint analysis based on p-value using PDA. Throughout this paper, we set the host name of working directory to be 'C:\Program Files\MATLAB71\PDA'. All input data files for these four examples are available with software PDA and saved in the example directory, 'C:\Program Files\MATLAB71\PDA\Example'.

#### Example 1: one-group single-point analysis

We used the six SNP data published in our previous paper [11] to illustrate the one-group analysis, the purpose being to estimate allele frequency. The operation procedures are illustrated in Appendix F (See Additional File 6).

Table 1 and Table 2 present the results from PDA for the six SNPs. Table 1 shows the estimated results for CPA. The 1^st ^column shows the SNP number. The 2^nd ^column shows the SNP name. The 3^rd ^column shows the number of heterozygous individuals. Three discrete adjustments (κ^H
 MathType@MTEF@5@5@+=feaafiart1ev1aaatCvAUfKttLearuWrP9MDH5MBPbIqV92AaeXatLxBI9gBaebbnrfifHhDYfgasaacH8akY=wiFfYdH8Gipec8Eeeu0xXdbba9frFj0=OqFfea0dXdd9vqai=hGuQ8kuc9pgc9s8qqaq=dirpe0xb9q8qiLsFr0=vr0=vr0dc8meaabaqaciaacaGaaeqabaqabeGadaaakeaaiiGacuWF6oWAgaqcamaaBaaaleaacqqGibasaeqaaaaa@2FB8@, κ^U
 MathType@MTEF@5@5@+=feaafiart1ev1aaatCvAUfKttLearuWrP9MDH5MBPbIqV92AaeXatLxBI9gBaebbnrfifHhDYfgasaacH8akY=wiFfYdH8Gipec8Eeeu0xXdbba9frFj0=OqFfea0dXdd9vqai=hGuQ8kuc9pgc9s8qqaq=dirpe0xb9q8qiLsFr0=vr0=vr0dc8meaabaqaciaacaGaaeqabaqabeGadaaakeaaiiGacuWF6oWAgaqcamaaBaaaleaacqqGvbqvaeqaaaaa@2FD2@) are shown along with the corresponding s.e. For example, for the 6^th ^SNP with SNP name 639, there are 36 heterozygous individuals used to calculate the CPA adjustment. The arithmetic mean adjustment is 2.288, with s.e. 0.038; the unbiased adjustment is 2.320, with s.e. 0.006; the geometric adjustment is 2.265, with s.e. 0.009.

In Table 2, PDA provides the allele frequency estimates. The 1^st ^column shows the SNP number. The 2^nd ^column shows the SNP name. The 3^rd ^panel shows the unadjusted allele frequencies and the corresponding s.e. The 4^th^, 5^th ^and 6^th ^panels show the allele frequency estimates based on the three adjustments (κ^H
 MathType@MTEF@5@5@+=feaafiart1ev1aaatCvAUfKttLearuWrP9MDH5MBPbIqV92AaeXatLxBI9gBaebbnrfifHhDYfgasaacH8akY=wiFfYdH8Gipec8Eeeu0xXdbba9frFj0=OqFfea0dXdd9vqai=hGuQ8kuc9pgc9s8qqaq=dirpe0xb9q8qiLsFr0=vr0=vr0dc8meaabaqaciaacaGaaeqabaqabeGadaaakeaaiiGacuWF6oWAgaqcamaaBaaaleaacqqGibasaeqaaaaa@2FB8@, κ^U
 MathType@MTEF@5@5@+=feaafiart1ev1aaatCvAUfKttLearuWrP9MDH5MBPbIqV92AaeXatLxBI9gBaebbnrfifHhDYfgasaacH8akY=wiFfYdH8Gipec8Eeeu0xXdbba9frFj0=OqFfea0dXdd9vqai=hGuQ8kuc9pgc9s8qqaq=dirpe0xb9q8qiLsFr0=vr0=vr0dc8meaabaqaciaacaGaaeqabaqabeGadaaakeaaiiGacuWF6oWAgaqcamaaBaaaleaacqqGvbqvaeqaaaaa@2FD2@, κ^G
 MathType@MTEF@5@5@+=feaafiart1ev1aaatCvAUfKttLearuWrP9MDH5MBPbIqV92AaeXatLxBI9gBaebbnrfifHhDYfgasaacH8akY=wiFfYdH8Gipec8Eeeu0xXdbba9frFj0=OqFfea0dXdd9vqai=hGuQ8kuc9pgc9s8qqaq=dirpe0xb9q8qiLsFr0=vr0=vr0dc8meaabaqaciaacaGaaeqabaqabeGadaaakeaaiiGacuWF6oWAgaqcamaaBaaaleaacqqGhbWraeqaaaaa@2FB6@) along with their respective s.e. values. For example, the unadjusted allele frequency of the 1^st ^allele of SNP 639 is 0.806 (the allele frequency of the 2^nd ^allele is 0.194), and the s.e. is 0.051. After applying CPA adjustments, the accurate allele frequency estimate is about 0.64 and s.e. is 0.06. Three different adjustments yield similar results. In this example, there is a serious overestimation of allele frequency if the CPA adjustment is ignored.

#### Example 2: two-group single-point analysis

In this example, we analyze the data set from our previous project that compared the allele distributions of three main Taiwan subgroups in the human major histocompatibility complex (MHC) region. We selected two subgroups (Hakka and Han groups) and 4 SNPs for the illustrations. The operation procedures are illustrated in Appendix F (See Additional File 6).

The results are shown in Tables 3, 4, 5. Table 3 shows the CPA estimates along with the s.e. values for these four SNPs. The unbiased CPA estimates are 1.68, 1.60, 1.39 and 1.77, and the corresponding s.e. values are 0.013, 0.010, 0.009 and 0.007.

Table 4 shows the allele frequency estimates along with s.e. Based on the unbiased adjustment of CPA, the allele frequency estimates (s.e. values) of SNPs 6260, 6267, 6272 and 6415 in the Hakka group are 0.93 (0.013), 0.94 (0.012), 0.60 (0.024) and 0.13 (0.016), respectively. The allele frequency estimates (s.e. values) of SNPs in the Han group are 0.84 (0.018), 0.82 (0.019), 0.62 (0.024) and 0.19 (0.019), respectively.

In Table 5, PDA conducted association tests using the four SNPs to compare the allele distributions between Hakka and Han groups. Firstly, the association test without applying CPA adjustment was conducted. The chi square statistic and the corresponding p-value were calculated for each SNP. Secondly, modified association statistics *X *based on the three different CPA adjustments were conducted. The s.e. of experimental error was set to be 0.02 according to our previous study [8]. For example, the association test based on the unbiased adjustment yields chi square statistics 5.54, 11.51, 0.23 and 2.95 and p-values 0.019, 0.001, 0.634 and 0.086 respectively. The conclusions from the unadjusted association test and adjusted association test are quite different.

In our previous project, these four SNPs were also genotyped individually and the allele-based association test based on individual genotyping data yielded the exact p-values for these four SNPs are 0.00795, 0.00006, 0.52346 and 0.23972 respectively. The conclusions are consistent with the results from the adjusted association tests and demonstrate the importance of CPA adjustment.

#### Example 3: two-group multipoint analysis based on peak intensity data

In this example, we illustrate a multipoint analysis, an important utility of PDA. We analyzed 10 SNPs from our MHC study to screen for potential candidate regions that could distinguish Hakka and Han groups. The operation procedures are illustrated in Appendix F (See Additional File 6).

The results are shown in Tables 6, 7, 8, 9. Table 6 shows the CPA estimates along with s.e. values for the ten SNPs. Table 7 shows the allele frequency estimates along with s.e. values. Table 8 shows the single-point pooled DNA association tests comparing the allele distributions between Hakka and Han groups. The results show that only SNP 6419 is significant; the p-value is 0.019 for the statistic without adjusting CPA, whereas it is 0.025 after adjusting CPA.

**Figure 2:**
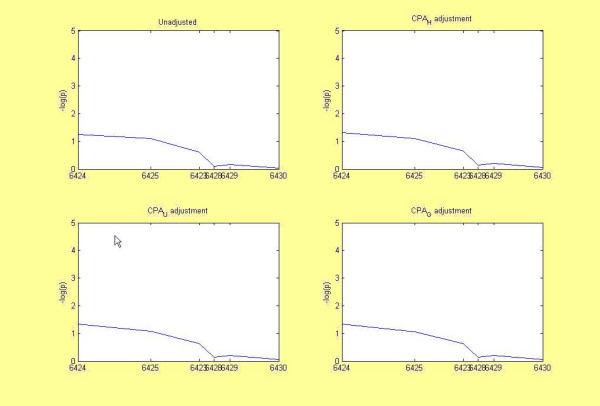
The transformed p-values of multiplicative SWEPT statistic based on different CPAs by using peak intensity data in Example 3.

Table 9 shows the multipoint pooled DNA association tests. The results firstly describe the input information of the analysis. In this example, peak intensity data, map information and equal weight were considered in the analysis, and the p-value was not truncated. We carried out 10000 Monte Carlo simulations to calculate the empirical p-value. The size of each window was 5, and a multiplicative p-value statistic was used. Using these settings, multipoint tests based on different CPAs were conducted. The results also are presented in Figure 2, where p-values were transformed by taking the minus log 10. For example, based on the unbiased adjustment of CPA, the p-values for the six sliding windows (with window size 5) are 0.047, 0.84, 0.229, 0.718, 0.629 and 0.874.

In our previous project, these ten SNPs were also genotyped individually, and the allele-based association test based on individual genotyping data yielded exact p-values for these ten SNPs: 0.0216, 0.0052, 0.0115, 0.6859, 0.0232, 0.9440, 0.1628, 0.4468, 0.4082 and 0.9443. However, the previous single-point pooled DNA test only identified SNP 6419. In this case, the important SNPs, 6421 and 6422, were not identified by the single-point association tests; however, the two SNPs are included in the region from SNPs 6421 to 6424, which was identified by a multipoint analysis based on a sliding window with size 5.

#### Example 4: two-group multipoint analysis based on p-value data

In this example, we illustrate the implementation of p-value analysis using PDA. To conduct multipoint association tests, we used the same 10 SNPs as in Example 3, based on the p-value derived from a single-point pooled DNA association test with unbiased adjustment of CPA. The operation procedures are illustrated in Appendix F (See Additional File 6).

Because we only implemented the p-value of each SNP, the procedures for the CPA estimate, allele frequency estimate and single-point association test cannot be considered in the analysis. Only multipoint association tests can be conducted.

**Figure 3:**
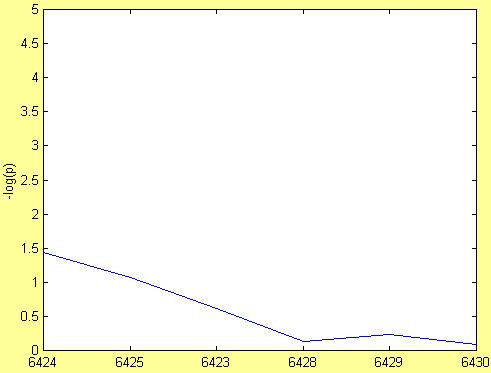
The transformed p-values of multiplicative SWEPT statistic using p-value data in Example 4.

First, PDA shows the input information for the analysis in this example, as follows: p-value data were used; no map information was provided; user-specified weights were used; the threshold value of truncation was 1; the number of Monte Carlo simulations was 10000; the size of each window was 5; the SWEPT statistic was calculated using the additive model. The results are summarized in Table 10 and are presented in Figure 3. Table 10 shows the SWEPT statistics and p-values for the six regions, each of which contains five SNPs. Because the same SNP data were used in Examples 3 and 4, it is not surprising that the results are similar to those in Example 3.

## Discussion

CPA estimation is based on peak intensity data of heterozygous individuals. Data of heterozygous individuals in a pilot study may not be available occasionally. Public accessible CPA databases for SNPs provide important information [25,26]. PDA allows for allele frequency estimation and association testing by directly inputting CPA values of SNPs of interest. This function enhances PDA to analyze large numbers of SNPs on the public databases in pooled DNA analysis.

PDA provides an extended single-point association test allowing for different CPAs between two comparative groups. This test reduces to the conventional test in [11] if the equal CPA between two groups is assumed. If typing of case and control DNA pools is performed at the same time under the same experimental conditions, then the reduced test should be applied. However, if the DNA pools of case and control groups are typed under different time/environments, e.g., a meta analysis and a sequential analysis, then the extended test should be performed.

Haplotype-scoring [27] and locus-scoring approaches [28] are the two main categories of association tests for disease gene mapping; however, it is currently unclear as to which method is superior while analysing individual genotyping data. We first introduce locus-scoring approach to analyze pooled DNA data. The SWEPT method considered in PDA is a locus-scoring approach, which does not require an inference to phase-unknown haplotypes; hence the locus-scoring approach has several advantages, among which is the reduction of computational burden. Until a breakthrough in economic efficiencies of haplotyping, locus-scoring approach is preferred than haplotype-scoring approach while performing pooled DNA analyses.

Weights for different SNPs in each window may affect the significance of a multipoint association test. If there is no prior knowledge in this regard, then equal weights can be employed. The other strategy is to consider weights according to genetic/physical or linkage disequilibrium maps of SNPs [29]. Using information of haplotype maps to improve the estimation of allele frequency difference at each single locus for association mapping has been considered in [30]. In our method, a SNP should be assigned a higher weight if the SNP marker is closer to the anchor in the center of a window. Anchors scan over the chromosome region of interest simultaneously when sliding windows move from the start to the end of all SNPs.

The sliding window procedure emphasizes a local effect, which assumes the neighboring SNPs provide sufficient information for the window of interest and that other SNPs outside the window do not impact the inference of the window once SNPs within the window have been considered. A small proportion of SNPs is considered each time, making the sliding-window approach a convenient and practical procedure for chromosome-wide studies once the window size is determined. A sliding-window size of 5 for the selection of genetic markers for association tests with individual genotyping data was suggested in [31], but they warned that this value might not be suitable in certain situations. We suggest that genetic background of studied region should be considered and several window sizes about the size of 5 should be analyzed to yield reliable results.

## Conclusion

PDA provides simultaneous analyses of the CPA adjustment, adjusted allele frequency estimate and single/multipoint DNA pooling association tests that are usually essential for complete DNA pooling studies. All of the PDA functions are illustrated in the four bona fide examples contained in the program. PDA is simple to operate and does not require that users have a strong statistical background.

## Availability and requirements

PDA software can be downloaded from the web site: .

**Project name: **DNA pooling project

**Project home page: **

**Operating system: **MS Windows^®^

**Programming language: **MATLAB^®^

**Other requirements: **No

**License: **PDA license

**Any restrictions to use by non-academics: **On request and citation

## Abbreviations

PDA: Pooled DNA analyzer

CPA: Coefficient of preferential amplification

SWEPT: Sliding-window empirical p-value test

## Authors' contributions

HCY conceived the statistical methods and experimental designs and prepared the manuscript. CCP programmed the software. CYL and CSJF contributed to the discussion and preparation of the manuscript. All authors have approved the final manuscript.

## Supplementary Material

Additional File 1 Appendix A – Installation and initialization of PDA Click here for file

Additional File 2 Appendix B – Description of working directories Click here for file

Additional File 3 Appendix C – Data input format Click here for file

Additional File 4 Appendix D – Results output format Click here for file

Additional File 5 Appendix E – Execution of PDA without MATLAB^®^ Click here for file

Additional File 6 Appendix F – Operation procedures in examples Click here for file

## Figures and Tables

**Figure 4:**
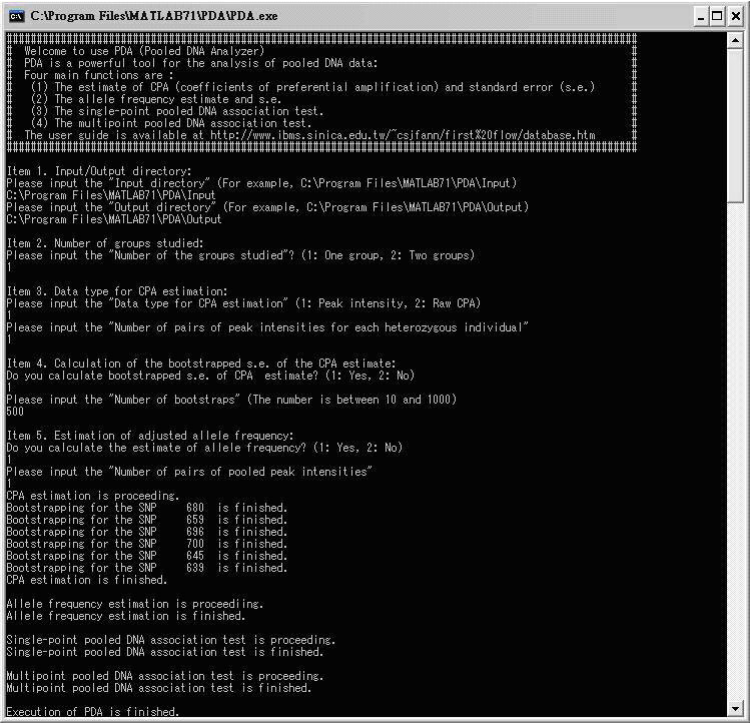
Interface of the execution of PDA on machines without MATLAB^® ^installed.

**Table 1:** The analysis of one-group DNA pooling data in Example 1 (Part 1: The CPA estimate and standard error (s.e.))

OBS	SNP	N_h	CPA_H	s.e.	CPA_U	s.e.	CPA_G	s.e.
1	680	13	1.259	0.032	1.221	0.014	1.252	0.022
2	659	40	0.765	0.052	0.674	0.040	0.687	0.041
3	696	42	0.662	0.032	0.632	0.032	0.634	0.032
4	700	34	0.873	0.009	0.851	0.007	0.859	0.008
5	645	44	1.771	0.007	1.788	0.006	1.749	0.007
6	639	36	2.288	0.038	2.320	0.006	2.265	0.009

**Table 2:** The analysis of one-group DNA pooling data in Example 1 (Part 2: The allele frequency estimate (AFE) and standard error (s.e.))

		Unadjusted		CPA_H adjustment		CPA_U adjustment		CPA_G adjustment	
OBS	SNP	AFE	s.e.	AFE	s.e.	AFE	s.e.	AFE	s.e.

1	680	0.945	0.029	0.932	0.033	0.934	0.032	0.932	0.032
2	659	0.034	0.024	0.044	0.027	0.050	0.028	0.049	0.028
3	696	0.163	0.048	0.228	0.054	0.236	0.055	0.235	0.055
4	700	0.643	0.062	0.673	0.061	0.679	0.060	0.677	0.060
5	645	0.817	0.050	0.716	0.058	0.714	0.058	0.719	0.058
6	639	0.806	0.051	0.644	0.062	0.641	0.062	0.647	0.062

**Table 3:** The analysis of two-group DNA pooling data in Example 2 (Part 1: The CPA estimate and standard error (s.e.))

OBS	SNP	N_h	CPA_H	s.e.	CPA_U	s.e.	CPA_G	s.e.
1	6260	18	1.699	0.016	1.683	0.013	1.685	0.015
2	6267	10	1.643	0.127	1.597	0.010	1.638	0.012
3	6272	48	1.400	0.010	1.388	0.009	1.379	0.009
4	6415	16	1.880	0.186	1.770	0.007	1.858	0.014

**Table 4:** The analysis of two-group DNA pooling data in Example 2 (Part 2: The allele frequency estimate (AFE) and standard error (s.e.))

The first group									
		Unadjusted		CPA_H adjustment		CPA_U adjustment		CPA_G adjustment	

OBS	SNP	AFE	s.e.	AFE	s.e.	AFE	s.e.	AFE	s.e.

1	6260	0.954	0.010	0.924	0.013	0.925	0.013	0.925	0.013
2	6267	0.961	0.009	0.937	0.012	0.939	0.012	0.938	0.012
3	6272	0.671	0.023	0.593	0.024	0.595	0.024	0.596	0.024
4	6415	0.207	0.020	0.122	0.016	0.128	0.016	0.123	0.016

The second group									

		Unadjusted		CPA_H adjustment		CPA_U adjustment		CPA_G adjustment	

OBS	SNP	AFE	s.e.	AFE	s.e.	AFE	s.e.	AFE	s.e.

1	6260	0.899	0.015	0.839	0.018	0.840	0.018	0.840	0.018
2	6267	0.877	0.016	0.813	0.019	0.817	0.019	0.813	0.019
3	6272	0.690	0.023	0.614	0.024	0.616	0.024	0.617	0.024
4	6415	0.298	0.022	0.184	0.019	0.194	0.019	0.186	0.019

**Table 5:** The analysis of two-group DNA pooling data in Example 2 (Part 3: The single-point DNA-pooling association test)

		Unadjusted		CPA_H adjustment		CPA_U adjustment		CPA_G adjustment	
OBS	SNP	chi^2	p-value	chi^2	p-value	chi^2	p-value	chi^2	p-value

1	6260	2.726	0.099	5.605	0.018	5.540	0.019	5.547	0.019
2	6267	6.136	0.013	11.465	0.001	11.507	0.001	11.875	0.001
3	6272	0.199	0.656	0.228	0.633	0.227	0.634	0.227	0.634
4	6415	4.931	0.026	2.727	0.099	2.946	0.086	2.798	0.094

**Table 6:** The multipoint analysis using peak intensity data in Example 3 (Part 1: The CPA estimate and standard error (s.e.))

OBS	SNP	N_h	CPA_H	s.e.	CPA_U	s.e.	CPA_G	s.e.
1	6421	38	2.660	0.002	2.644	0.003	2.641	0.003
2	6422	37	1.772	0.003	1.762	0.002	1.755	0.003
3	6419	36	1.704	0.003	1.689	0.002	1.689	0.002
4	6409	12	1.348	0.003	1.331	0.003	1.339	0.003
5	6424	41	1.797	0.004	1.783	0.003	1.784	0.003
6	6425	37	1.759	0.250	1.655	0.168	1.699	0.188
7	6423	4	1.981	0.004	1.994	0.004	1.979	0.005
8	6428	38	2.041	0.003	2.023	0.003	2.024	0.003
9	6429	38	1.768	0.003	1.751	0.003	1.754	0.003
10	6430	37	1.586	0.002	1.575	0.002	1.579	0.002

**Table 7:** The multipoint analysis using peak intensity data in Example 3 (Part 2: The allele frequency estimate (AFE) and standard error (s.e.))

The first group									
		Unadjusted		CPA_H adjustment		CPA_U adjustment		CPA_G adjustment	

OBS	SNP	AFE	s.e.	AFE	s.e.	AFE	s.e.	AFE	s.e
1	6421	0.816	0.019	0.626	0.024	0.627	0.024	0.627	0.024
2	6422	0.505	0.024	0.365	0.023	0.367	0.024	0.368	0.024
3	6419	0.457	0.024	0.330	0.023	0.332	0.023	0.332	0.023
4	6409	0.935	0.012	0.914	0.014	0.915	0.014	0.915	0.014
5	6424	0.649	0.023	0.507	0.024	0.509	0.024	0.509	0.024
6	6425	0.754	0.021	0.636	0.023	0.650	0.023	0.644	0.023
7	6423	0.974	0.008	0.949	0.011	0.949	0.011	0.949	0.011
8	6428	0.531	0.024	0.357	0.023	0.359	0.023	0.359	0.023
9	6429	0.651	0.023	0.514	0.024	0.516	0.024	0.516	0.024
10	6430	0.668	0.023	0.559	0.024	0.560	0.024	0.560	0.024

The second group									

		Unadjusted		CPA_H adjustment		CPA_U adjustment		CPA_G adjustment	

OBS	SNP	AFE	s.e.	AFE	s.e.	AFE	s.e.	AFE	s.e.

1	6421	0.776	0.020	0.566	0.024	0.567	0.024	0.568	0.024
2	6422	0.589	0.024	0.447	0.024	0.448	0.024	0.449	0.024
3	6419	0.561	0.024	0.428	0.024	0.431	0.024	0.430	0.024
4	6409	0.927	0.013	0.904	0.014	0.905	0.014	0.905	0.014
5	6424	0.590	0.024	0.445	0.024	0.447	0.024	0.446	0.024
6	6425	0.733	0.022	0.609	0.024	0.624	0.024	0.618	0.024
7	6423	0.968	0.009	0.938	0.012	0.937	0.012	0.938	0.012
8	6428	0.543	0.024	0.368	0.024	0.370	0.024	0.369	0.024
9	6429	0.682	0.023	0.548	0.024	0.550	0.024	0.550	0.024
10	6430	0.656	0.023	0.546	0.024	0.548	0.024	0.548	0.024

**Table 8:** The multipoint analysis using peak intensity data in Example 3 (Part 3: The single-point pooled DNA association test)

		Unadjusted		CPA_H adjustment		CPA_U adjustment		CPA_G adjustment	
OBS	SNP	chi^2	p-value	chi^2	p-value	chi^2	p-value	chi^2	p-value

1	6421	1.034	0.309	1.847	0.174	1.844	0.175	1.843	0.175
2	6422	3.533	0.060	3.399	0.065	3.405	0.065	3.408	0.065
3	6419	5.466	0.019	5.016	0.025	5.032	0.025	5.031	0.025
4	6409	0.052	0.820	0.080	0.778	0.078	0.780	0.079	0.779
5	6424	1.799	0.180	1.949	0.163	1.950	0.163	1.950	0.163
6	6425	0.272	0.602	0.368	0.544	0.360	0.549	0.363	0.547
7	6423	0.041	0.840	0.127	0.722	0.128	0.720	0.127	0.722
8	6428	0.065	0.799	0.059	0.809	0.059	0.808	0.059	0.808
9	6429	0.494	0.482	0.580	0.446	0.580	0.446	0.580	0.446
10	6430	0.067	0.796	0.077	0.782	0.077	0.782	0.077	0.782

**Table 9:** The multipoint analysis using peak intensity data in Example 3 (Part 4: The multipoint pooled DNA association test)

SNP		Unadjusted		CPA_H adjustment		CPA_U adjustment		CPA_G adjustment	
Start	End	SWEPT	p-value	SWEPT	p-value	SWEPT	p-value	SWEPT	p-value

6421	6424	0.474	0.058	0.633	0.048	0.578	0.047	0.335	0.046
6422	6425	0.474	0.080	0.480	0.078	0.722	0.084	0.630	0.086
6419	6423	0.279	0.242	0.510	0.228	0.330	0.229	0.620	0.229
6409	6428	0.216	0.786	0.531	0.719	0.341	0.718	0.604	0.712
6424	6429	0.227	0.697	0.668	0.618	0.188	0.629	0.396	0.619
6425	6430	0.176	0.925	0.644	0.871	0.204	0.874	0.232	0.869

**Table 10:** The multipoint analysis using p-value data in Example 4

SNP		Test results	
Start	End	SWEPT	p-value

6421	6424	0.521	0.037
6422	6425	0.551	0.084
6419	6423	0.439	0.244
6409	6428	0.574	0.730
6424	6429	0.537	0.591
6425	6430	0.617	0.825

## References

[B1] Hirschhorn JN, Daly MJ (2005). Genome-wide association studies for common diseases and complex traits. Nat Rev Genet.

[B2] Wang WYS, Barratt BJ, Clayton DG, Todd JA (2005). Genome-wide association studies: The theoretical and practical concerns. Nat Rev.

[B3] Arnheim N, Strange C, Erlich H (1985). Use of pooled DNA samples to detect linkage disequilibrium of polymorphic restriction fragments and human disease: studies of the *HLA *class II loci. Proc Natl Acad Sci USA.

[B4] Mohlke KL, Erdos MR, Scott LJ, Fingerlin TE, Jackson AU, Silander K, Hollstein P, Boehnke M, Collins FS (2002). High-throughput screening for evidence of association by using mass spectrometry genotyping on DNA pools. Proc Natl Acad Sci USA.

[B5] Herbon N, Werner M, Braig C, Gohlke H, Dütsch G, Illig T, Altmüller J, Hampe J, Lantermann A, Schreiber S, Bonifacio E, Ziegler A, Schwab S, Wildenauer D, van den Boom D, Braun A, Knapp M, Reitmeir P, Wjst M (2003). High-resolution SNP scan of chromosome 6p21 in pooled samples from patients with complex diseases. Genomics.

[B6] Buetow KH, Edmonson M, MacDonald R, Clifford R, Yip P, Kelley J, Little DP, Strausberg R, Koester H, Cantor CR, Braun A (2001). High-throughput development and characterization of a genomewide collection of gene-based single nucleotide polymorphism markers by chip-based matrix-assisted laser desorption/ionization time-of-flight mass spectrometry. Proc Natl Acad Sci USA.

[B7] Nelson MR, Marnellos G, Kammerer S, Hoyal CR, Shi MM, Cantor CR, Braun A (2004). Large-scale validation of single nucleotide polymorphisms in gene regions. Genome Res.

[B8] Yang HC, Lin CH, Hung SI, Fann CSJ Polymorphism validation using DNA pools prior to conducting large-scale genetic studies. Ann Hum Genet.

[B9] Sham P, Bader JS, Craig I, O'Donovan M, Owen M (2002). DNA pooling: A tool for large-scale association studies. Nat Rev Genet.

[B10] Yang HC, Fann CSJ, Collins A (2006). Association mapping using pooled DNA. Linkage Disequilibrium and Association Mapping.

[B11] Yang HC, Pan CC, Lu RCY, Fann CSJ (2005). New adjustment factors and sample size calculation in a DNA-pooling experiment with preferential amplification. Genetics.

[B12] Hoogendoorn B, Norton N, Kirov G, Williams N, Hamshere ML, Spurlock G, Austin J, Stephens MK, Buckland PR, Owen MJ, O'Donovan MC (2000). Cheap, accurate and rapid allele frequency estimation of single nucleotide polymorphisms by primer extension and DHPLC in DNA pools. Hum Genet.

[B13] Visscher PM, Le Hellard S (2003). Simple method to analyze SNP-based association studies using DNA pools. Genet Epidemiol.

[B14] Ito T, Chiku S, Inoue E, Tomita M, Morisaki T, Morisaki H, Kamatani N (2003). Estimation of haplotype frequencies, linkage-disequilibrium measures, and combination of haplotype copies in each pool by use of pooled DNA data. Am J Hum Genet.

[B15] Wang S, Kidd KK, Zhao H (2003). On the use of DNA pooling to estimate haplotype frequencies. Genet Epidemiol.

[B16] Yang Y, Zhang J, Hoh J, Matsuda F, Xu P, Lathrop M, Ott J (2003). Efficiency of single-nucleotide polymorphism haplotype estimation from pooled DNA. Proc Natl Acad Sci USA.

[B17] Zeng D, Lin DY (2005). Estimating haplotype-disease associations with pooled genotype data. Genet Epidemiol.

[B18] Barratt BJ, Payne F, Rance HE, Nutland S, Todd JA, Clayton DG (2002). Identification of the sources of error in allele frequency estimations from pooled DNA indicates an optimal experimental design. Ann Hum Genet.

[B19] Downes K, Barratt BJ, Akan P, Bumpstead SJ, Taylor SD, Clayton DG, Deloukas P (2004). SNP allele frequency estimation in DNA pools and variance components analysis. Biotechniques.

[B20] Zaykin DV, Zhivotovsky LA, Westfall PH, Weir BS (2002). Truncated product method for combing p-values. Genet Epidemiol.

[B21] Edgington ES (1972). An additive model for combining probability values from independent experiments. J Psychol.

[B22] Zheng G (2003). Use of max and min scores for trend tests for association when the genetic model is unknown. Stat Med.

[B23] Yu K, Gu CC, Province M, Xiong CJ, Rao DC (2004). Genetic association mapping under founder heterogeneity via weighted haplotype similarity analysis in candidate genes. Genet Epidemiol.

[B24] Dudbridge F, Koeleman BPC (2003). Rank truncated product of p-values, with application to genomewide association scans. Genet Epidemiol.

[B25] Simpson CL, Knight J, Butcher LM, Hansen VK, Meaburn E, Schalkwyk LC, Craig IW, Powell JF, Sham PC, AL-Chalabi A (2005). A central resource for accurate allele frequency estimation from pooled DNA genotyped on DNA microarrays. Nucleic Acids Res.

[B26] The Database of Coefficient of Preferential Amplification/Hybridization. http://www.ibms.sinica.edu.tw/%7Ecsjfann/first%20flow/database.htm.

[B27] Morris RW, Kaplan NL (2002). On the advantage of haplotype analysis in the presence of multiple disease susceptibility alleles. Genet Epidemiol.

[B28] Seaman SR, Müller-Myhsok B (2005). Rapid simulation of p values for product methods and multiple-testing adjustment in association studies. Am J Hum Genet.

[B29] Yang HC, Lin CY, Fann CSJ (2005). A unified multilocus association test [abstract]. Am J Hum Genet.

[B30] Hinds DA, Seymour AB, Durham LK, Banerjee P, Ballinger DG, Milos PM, Cox DR, Thompson JF, Frazer KA (2004). Application of pooled genotyping to scan candidate regions for association with HDL cholesterol levels. Human Genomics.

[B31] Meng Z, Zaykin DV, Xu CF, Wagner M, Ehm MG (2003). Selection of genetic markers for association analyses, using linkage disequilibrium and haplotypes. Am J Hum Genet.

