# Supplementary material for: PDA: Pooled DNA analyzer
Source: BMC Bioinformatics. 2006 Apr 28;7:233. doi: 10.1186/1471-2105-7-233 (PMC1539032; doi:10.1186/1471-2105-7-233)
Supplement: Additional File 1 — Appendix A – Installation and initialization of PDA [file 1471-2105-7-233-S1.doc]

# Additional file 1

# Appendix A - Installation and initialization of PDA

PDA was developed using the software, MATLAB®, and can be run under two kinds of operation modes. This appendix illustrates the execution of PDA under MATLAB® environment, where MATLAB® must be installed before executing PDA. The execution of PDA under a stand-alone mode without installing MATLAB® is illustrated in Appendix E (See Additional file 5). The MATLAB® download and related information are available at http://www.mathworks.com/. Once MATLAB® is installed on your hard drive (e.g., ‘C:\Program Files\MATLAB71’ for version 7.0.1), PDA can be initialized following the procedures listed below (MATLAB® version 7.0.1 is illustrated in this appendix).

1. Download program PDA, a zip file ‘PDA.zip’, from the website http://www.ibms.sinica.edu.tw/%7Ecsjfann/first flow/pda.htm.
2. Unzip ‘PDA.zip’, and copy the directory ‘PDA’ to the designated directory, ‘C:\Program Files\MATLAB71’, where MATLAB® was installed.
3. Initialize MATLAB®, and enter the user interface.
4. Click the ‘File’ button in the command bar of ‘MATLAB Command Window’, and select the ‘Set Path’ button.
5. Click the ‘Add path’ button, and select the working directory, ‘PDA’, in the directory, ‘C:\Program Files\MATLAB71’, to add path. Click the ‘Save’ button to save the path.
6. Key in the command ‘PDA’ in the command line in ‘MATLAB Command Window’ to enter the PDA environment. The user interface of PDA is shown in Figure 1.
