# Supplementary material for: PDA: Pooled DNA analyzer
Source: BMC Bioinformatics. 2006 Apr 28;7:233. doi: 10.1186/1471-2105-7-233 (PMC1539032; doi:10.1186/1471-2105-7-233)
Supplement: Additional File 2 — Appendix B – Description of working directories [file 1471-2105-7-233-S2.doc]

# Additional file 2

# Appendix B - Description of working directories

About the working directories of PDA, the main directory name of PDA is ‘PDA’. There are four components in this directory, as follows:

1. Files with filename *.m are the program source codes of PDA.
2. Directory ‘Input’: All data analyzed by PDA MUST be saved in this directory.
3. Directory ‘Output’: All results will be saved automatically in this directory.
4. Directory ‘Example’: The data files of four examples for illustrating the use of PDA are saved in this directory.
