# Supplementary material for: PDA: Pooled DNA analyzer
Source: BMC Bioinformatics. 2006 Apr 28;7:233. doi: 10.1186/1471-2105-7-233 (PMC1539032; doi:10.1186/1471-2105-7-233)
Supplement: Additional File 3 — Appendix C – Data input format [file 1471-2105-7-233-S3.doc]

# Additional file 3

**Appendix C - Data input format**

Five data files are requested optionally to perform PDA. These files must be saved in the working directory, ‘C:\Program Files\MATLAB71\PDA\Input’.

(1) SNP name:

This file records the list of SNP names. The SNP names must be arranged in a column (i.e., one row per SNP) and MUST be Arabic numerals. The file must be saved with the filename, ‘SnpName.txt’. If users want to provide the information of marker position for a better graphic demonstration of final results of multipoint association tests, then marker position should be listed in the second column, followed by the SNP names.

(2) Peak intensity data of heterozygous individuals:

If ‘raw CPA/heterozygote ratio’ is checked in Item 3, then this file contains three columns; if ‘peak intensity’ is checked, then there are four columns at least. The file must be saved with the filename, ‘IndPI.txt’.

- - - 1. The 1^st^ column is the index of groups. Code 1 signifies the 1^st^ group, and code 2, the 2^nd^ group. If only one group is included in the analysis or constant CPA between different groups are assumed, then this column is a column vector containing only 1s.
      2. The 2^nd^ column is the SNP name. The order of SNPs must match the order in ‘SnpName.txt’.
      3. When ‘raw CPA/heterozygote ratio’ is selected for data type in Item 3, then the 3^rd^ column is CPA values. When ‘peak intensity’ is selected, then the 3^rd^ and 4^th^ columns are the peak intensities of the first and second alleles of heterozygous individuals, respectively. If more than one pair of peak intensities are inputted for CPA estimation, then they should be listed followed by the 4^th^ column.

(3) Unadjusted AF or peak intensity in DNA pools:

This file contains four columns at least and must be saved with the filename, ‘PoolAF.txt’.

1. The 1^st^ column is the index of groups. If unequal CPAs analysis is performed, then the labelling in this column must match with the first column in file ‘IndPI.txt’.
2. The 2^nd^ column is the SNP name. The order of SNPs must match the order in ‘SnpName.txt’.
3. The 3^rd^ column is the pool size.
4. The 4^th^ and 5^th^ columns are the unadjusted AF of the two alleles in the DNA pools. If more than one peak intensity pair is inputted, then they should be listed in order followed by the 5^th^ column.

(4) P-value:

This file contains two columns and must be saved with the filename, ‘Pvalue.txt’.

1. The 1^st^ column is the SNP name. The order of SNPs must match the order in ‘SnpName.txt’.
2. The 2^nd^ column is the p-value of each SNP from a previous single-point association test. It must be a real value between 0 and 1.

(5) Weight function:

This file contains two columns and must be saved with the filename, ‘Weight.txt’.

1. The 1^st^ column is the SNP name. The order of SNPs must match the order in ‘SnpName.txt’.
2. The 2^nd^ column is the weight of each SNP. It must be non-negative real values.

We will use four real examples in Section Results to illustrate uses of PDA. All input data files for these four examples are available with software PDA. Users can refer to these examples for the data input format.
