# Supplementary material for: PDA: Pooled DNA analyzer
Source: BMC Bioinformatics. 2006 Apr 28;7:233. doi: 10.1186/1471-2105-7-233 (PMC1539032; doi:10.1186/1471-2105-7-233)
Supplement: Additional File 4 — Appendix D – Results output format [file 1471-2105-7-233-S4.doc]

# Additional file 4

**Appendix D – Results output format**

PDA creates four output files with filenames, ‘CPA.txt’, ‘AF.txt’, ‘ASSO(single).txt’, ‘ASSO(multiple).txt’ and a LOG file with filename ‘Log.txt’, and will be save automatically in the destination directory, ‘C:\Program Files\MATLAB71\PDA\Output’.

When ‘peak intensity’ is checked in Item 3 for CPA estimation, the format of each file is explained below.

1. CPA.txt: The number and names of SNPs are shown first, followed by the number of heterozygous individuals and the estimates of CPA along with the corresponding s.e. values, based on three different methods (i.e., arithmetic mean adjustment, unbiased adjustment and geometric mean adjustment).
2. AF.txt: The number and name of SNP are shown first, followed by the unadjusted AF along with its s.e. The adjusted AF and its s.e., based on three different adjustments, are shown next.
3. ASSO(single).txt: The number and name of SNP are shown first, followed by the chi square statistic and p-value based on the unadjusted AF. Next, chi square statistics and p-values, based on three adjustments, are shown accordingly.
4. ASSO(multiple).txt: The information of seven options illustrated in Item 7 of Section Implementation is displayed first. Then, summary results are shown below. The names of start and end SNP are shown first, followed by the SWEPT test statistics and p-values.
5. Log.txt: This file records the running message for each SNP.

When ‘raw CPA/heterozygote ratio’ is checked in Item 3, the format of each file is explained below.

1. CPA.txt: The number and name of SNP are shown first, followed by the inputted CPA values.
2. AF.txt: The number and name of SNP are shown first, followed by the AF adjusted using the inputted CPA and the corresponding s.e.
3. ASSO(single).txt: The number and name of SNP are shown first, followed by the chi square statistic and p-value based on the unadjusted AF and the AF adjusted using the inputted CPA, in order.
4. ASSO(multiple).txt: The information of seven options illustrated in Item 7 of Section Implementation is displayed first. Then, summary results are shown below. The names of start and end SNP are shown first, followed by the SWEPT test statistics and p-values.
5. Log.txt: This file records the running message for each SNP.
