# Supplementary material for: PDA: Pooled DNA analyzer
Source: BMC Bioinformatics. 2006 Apr 28;7:233. doi: 10.1186/1471-2105-7-233 (PMC1539032; doi:10.1186/1471-2105-7-233)
Supplement: Additional File 5 — Appendix E – Execution of PDA without MATLAB® [file 1471-2105-7-233-S5.doc]

# Additional file 5

**Appendix E – Execution of PDA without MATLAB®**

For users who have no access to or little knowledge of the MATLAB® system, we used the MATLAB® compiler to generate standalone executables of PDA, which can be deployed on machines without installing the MATLAB®. Users can refer to the website: http://www.mathworks.com/access/helpdesk/help/toolbox/compiler/rn/bqnylfk-4.html for the compiling directions. Before running the executable files generated from the MATLAB® compiler, it is necessary to download and install the latest MATLAB® Component Runtime (MCR) Libraries.

The MCR (‘MCRinstaller.exe’) is freely available from the authors and can be installed by directly executing ‘MCRInstaller.exe’. All the necessary files of the compiled version of PDA are compressed (zipped) into an archive ‘PDA-MCR.zip’ and can be downloaded from the web site: http://www.ibms.sinica.edu.tw/%7Ecsjfann/first%20flow/pda.htm. After the installation of MCR, we can initialize PDA by executing ‘PDA-MCR.exe’. The interface accompanying with the demonstration of Example 1 is shown in Figure 4. The operation procedures of the compiled PDA version are the same with that of the graphical version (Figure 1) except that the former gets input data by manually keying them instead of clicking the checkboxes.
