# Supplementary material for: PDA: Pooled DNA analyzer
Source: BMC Bioinformatics. 2006 Apr 28;7:233. doi: 10.1186/1471-2105-7-233 (PMC1539032; doi:10.1186/1471-2105-7-233)
Supplement: Additional File 6 — Appendix F – Operation procedures in examples [file 1471-2105-7-233-S6.doc]

# Additional file 6

**Appendix F – Operation procedures in examples**

**The operation procedures of Example 1:**

1. Copy files ‘Example1(SnpName).txt’, ‘Example1(IndPI).txt’ and ‘Example1 (PoolAF).txt’ from the example directory, ‘C:\Program Files\MATLAB71\PDA\Example’, to the input directory, ‘C:\Program Files\MATLAB71\PDA\Input’, and rename the filenames as ‘SnpName.txt’, ‘IndPI.txt’ and ‘PoolAF.txt’, respectively.
2. Key in the command ‘PDA’ in the command line in ‘MATLAB Command Window’ to enter the PDA environment.
3. Check ‘One group’ for the query ‘Number of the groups studied?’
4. Check ‘Peak intensity’ for the query ‘Data type for CPA estimation?’ and key in ‘1’ for ‘Number of pairs of peak intensities for each heterozygous individual’.
5. Check ‘Yes’ for the query ‘Do you require the bootstrapped s.e. for CPA adjustments?’, and key in ‘500’ for ‘Number of bootstraps’.
6. Check ‘Yes’ for the query ‘Do you require the estimate of allele frequency?’ and key in ‘1’ for ‘Number of pairs of pooled peak intensities’.
7. Check ‘No’ for the query ‘Do you require the single-point pooled DNA association test?’
8. Check ‘No’ for the query ‘Do you require the multipoint pooled DNA association test?’
9. Click ‘Apply’ to submit job and please wait a while for execution of PDA.

**The operation procedures of Example 2:**

1. Copy files ‘Example2(SnpName).txt’, ‘Example2(IndPI).txt’ and ‘Example2 (PoolAF).txt’ from the example directory, ‘C:\Program Files\MATLAB71\PDA\Example’, to the input directory, ‘C:\Program Files\MATLAB71\PDA\Input’, and rename the filenames as ‘SnpName.txt’, ‘IndPI.txt’ and ‘PoolAF.txt’, respectively.
2. Key in the command ‘PDA’ in the command line in ‘MATLAB Command Window’ to enter the PDA environment.
3. Check ‘Two groups’ for the query ‘Number of the groups studied?’ and ‘Yes’ for the query ‘Assume constant CPA between different groups’.
4. Check ‘Peak intensity’ for the query ‘Data type for CPA estimation?’ and key in ‘1’ for ‘Number of pairs of peak intensities for each heterozygous individual’.
5. Check ‘Yes’ for the query ‘Do you require the bootstrapped s.e. for CPA adjustments?’ and key in ‘500’ for ‘Number of bootstraps’.
6. Check ‘Yes’ for the query item ‘Do you require the estimate of allele frequency?’ and key in ‘1’ for ‘Number of pairs of pooled peak intensities’.
7. Check ‘Yes’ for the query item ‘Do you require the single-point pooled DNA association test?’ and assign 0.02 for the query ‘Experimental error?’
8. Check ‘No’ for the query ‘Do you require the multipoint pooled DNA association test?’
9. Click ‘Apply’ to submit the job. The execution of PDA will require a waiting period.

**The operation procedures of Example 3:**

1. Copy files ‘Example3(SnpName).txt’, ‘Example3(IndPI).txt’ and ‘Example3(PoolAF).txt’ from the example directory, ‘C:\Program Files\MATLAB71\PDA\Example’, to the input directory, ‘C:\Program Files\MATLAB71\PDA\Input’, and rename the filenames as ‘SnpName.txt’, ‘IndPI.txt’ and ‘PoolAF.txt’, respectively.
2. Key in the command ‘PDA’ in the command line in ‘MATLAB Command Window’ to enter the PDA environment.
3. Check ‘Two groups’ for the query ‘Number of the groups studied?’ and ‘Yes’ for the query ‘Assume constant CPA between different groups’.
4. Check ‘Peak intensity’ for the query ‘Data type for CPA estimation?’ and key in ‘1’ for ‘Number of pairs of peak intensities for each heterozygous individual’.
5. Check ‘Yes’ for the query ‘Do you require the bootstrapped s.e. for CPA adjustments?’ and key in ‘500’ for ‘Number of bootstraps’.
6. Check ‘Yes’ for the query item ‘Do you require the estimate of allele frequency?’ and key in ‘1’ for ‘Number of pairs of pooled peak intensities’.
7. Check ‘Yes’ for the query item ‘Do you require the single-point pooled DNA association test?’ and assign 0.02 for the query ‘Experimental error?’
8. Check ‘Yes’ for the query item ‘Do you require the multipoint pooled DNA association test?’. Check ‘Yes’ for the query item ‘Map information’, check ‘Peak intensity’ to specify the data type, check ‘Equal weight’ to consider weight for different SNPs, assign 1.00 for the query ‘Threshold value of truncation’, assign 10000 for the query ‘Number of Monte Carlo simulation’, assign 5 for the query ‘Window size’ and check ‘Multiplicative effect’ to use the multiplicative SWEPT statistic.
9. Click ‘Apply’ to submit the job. The execution of PDA will require a waiting period.

**The operation procedures of Example 4:**

1. Copy files ‘Example4(SnpName).txt’, ‘Example4(Pvalue).txt’ and ‘Example4(Weight).txt’ from the example directory, ‘C:\Program Files\MATLAB71\PDA\Example’, to the input directory, ‘C:\Program Files\MATLAB71\PDA\Input’, and rename the filenames as ‘SnpName.txt’, ‘Pvalue.txt’ and ‘Weight.txt’, respectively.
2. Key in the command ‘PDA’ in the command line in ‘MATLAB Command Window’ to enter the PDA environment.
3. Because only p-values are available, the 2^nd^ – 6^th^ queries are skipped.
4. Check ‘Yes’ for the query item ‘Do you require the multipoint pooled DNA association test?’. Check ‘P-value’ to specify the data type, check ‘No’ for the query item ‘Map information’, check ‘User-specified weight’ to consider weight for different SNPs, assign 1.00 for the query ‘Threshold value of truncation’, assign 10000 for the query ‘Number of Monte Carlo simulation’, assign 5 for the query ‘Window size’ and check ‘Multiplicative effect’ to use the multiplicative SWEPT statistic.
5. Click ‘Apply’ to submit the job. The execution of PDA will require a waiting period.
